# Angiotensin-converting enzyme inhibitors reduce oxidative stress intensity in hyperglicemic conditions in rats independently from bradykinin receptor inhibitors

**DOI:** 10.3325/cmj.2016.57.371

**Published:** 2016-08

**Authors:** Kinga Mikrut, Justyna Kupsz, Jacek Koźlik, Hanna Krauss, Ewa Pruszyńska-Oszmałek, Magdalena Gibas-Dorna

**Affiliations:** 1 Department of Physiology, Poznan University of Medical Sciences, Poznań, Poland; 2 Department of Animal Physiology and Biochemistry, Poznan University of Life Sciences, Poznań, Poland

## Abstract

**Aim:**

To investigate whether bradykinin-independent antioxidative effects of angiotensin-converting enzyme inhibitors (ACEIs) exist in acute hyperglycemia.

**Methods:**

Male Wistar rats were divided into the normoglycemic group (n = 40) and the hyperglycemic group (n = 40). Hyperglycemia was induced by a single intraperitoneal injection of streptozotocin (STZ, 65 mg/kg body weight) dissolved in 0.1 mol/L citrate buffer (pH 4.5) 72 hours before sacrifice. The normoglycemic group received the same volume of citrate buffer. Each group was divided into five subgroups (n = 8): control group, captopril group, captopril + bradykinin B1 and B2 receptor antagonists group, enalapril group, and enalapril + bradykinin B1 and B2 receptor antagonists group. Captopril, enalapril, B1 and B2 receptor antagonists, or 0.15 mol/L NaCl were given at 2 and 1 hour before sacrifice. Oxidative status was determined by measuring the concentration of malondialdehyde and H_2_O_2_, and the activity of superoxide dismutase (SOD), catalase (CAT), and glutathione peroxidase (GPx).

**Results:**

In STZ-induced hyperglycemic rats ACEIs significantly reduced H_2_O_2_ and MDA concentration, while they significantly enhanced SOD and GPx activity. The hyperglycemic group treated simultaneously with ACEIs and bradykinin B1 and B2 receptor antagonists showed a significant decrease in H_2_O_2_ concentration compared to the control hyperglycemic group.

**Conclusion:**

These results suggest the existence of additional antioxidative effect of ACEIs in hyperglycemic conditions, which is not related to the bradykinin mediation and the structure of the drug molecule.

Hyperglycemia is a predominant pathogenic factor in micro- and macrovascular complications in diabetes mellitus (DM). However, there is evidence that acute glucose fluctuations have a greater impact on oxidative tissue damage in DM than sustained hyperglycemia (1). Hyperglycemia induces mitochondrial superoxide overproduction, leading to the activation of the consecutive sources of reactive oxygen, such as nicotinamide adenine dinucleotide phosphate oxidases (NADPH oxidases), uncoupled endothelial nitric oxide synthase (eNOS), protein kinase C isoforms, polyol and hexosamine pathways, as well as the increased formation of advanced glycation end products (AGEs) and stress-activated proteins including nuclear factor-κB (NF-κB), p38 kinase activated by mitogen (p38 MAPK), NH2-terminal Jun kinases/stress-activated protein kinases (JNK/SAPK), and Janus kinase/signal transducer and activator of transcription (JAK/STAT). In addition, hyperglycemia impairs the endogenous antioxidant defense system (2-4). This imbalance between radical-generating and radical-scavenging processes is an important factor in the mechanism of diabetic tissue damage. Considerable experimental and clinical evidence indicates a role of the renin-angiotensin system (RAS) in the pathogenesis of DM (5,6). It has been shown in both animal models and humans that DM is characterized by an elevated activity of angiotensin converting enzyme (ACE) (7,8). ACE converts angiotensin I (ANG-I) to angiotensin II (ANG-II), a potentially prooxidative agent, and simultaneously inactivates bradykinin, which is thought to have antioxidative properties. Accordingly, it can be assumed that ACE inhibition may play a certain role in the prevention of oxidative stress and DM development.

ACEIs are widely used in the treatment of cardiovascular diseases, especially hypertension, as well as atherosclerosis, myocardial infarction, and congestive heart failure. Additionally, as shown by several randomized trials, ACEIs and ANG-II receptor blockers (ARBs) are powerful agents minimizing the risk of DM (6,9). The majority of the beneficial effects of ACEIs result from the decrease in ANG-II concentration, increase in bradykinin bioavailability, and activation of intracellular bradykinin-dependent mechanisms (10,11). Bradykinin exerts physiologic effects through two types of G-protein-coupled receptors: type 2 (B2Rs) and type 1 (B1Rs). However, its biological action, including antioxidative activity, is mainly mediated through B2Rs. B1Rs are highly expressed or synthesized *de novo* under the influence of inflammatory factors, growth promoters, as well as hyperglycemia (12,13). Studies on a rat model of insulin resistance have shown that the B1Rs activation leads to the increased production of superoxide through NADPH oxidase (14). ACEIs can enhance both B1R and B2R signaling, acting as direct allosteric agonists of B1Rs, and as indirect allosteric enhancers of kinin B2Rs, via inactivation of ACE (15). Antioxidant effects of ACEIs are well known and widely accepted (10,16-18). Most studies suggest that this is the result of bradykinin action, however, ACEIs may also activate B1Rs and, thereby, enhance O2^●−^ production (19,20). Thus, the overall impact of ACEIs on oxidative processes has not been completely clarified yet. In this context, the aim of the study was to investigate whether bradykinin-independent antioxidative effects of ACEIs exist in streptozotocin (STZ)-induced acute hyperglycemia. Considering that both types of kinin receptors are involved in the regulation of the redox state, and that ACEIs affect their activity, we used B1 and B2 receptor antagonists to eliminate this pathway of ACEIs action.

## Methods

### Animals

**Figure 1:**
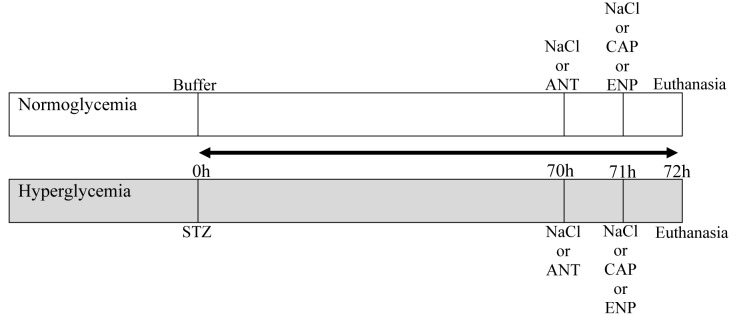
Experimental protocol. Streptozotocin (STZ), captopril (CAP), enalapril (ENP), bradykinin receptor antagonists (ANT).

All experimental and animal care procedures were conducted in accordance with the European Convention for the Protection of Vertebrate Animals Used for Experimental and Other Scientific Purposes (ETS 123/1986 and Appendix A/2006: Guidelines for accommodation and care of animals) and with the directive 2010/63/UE of the European Parliament and Council, as well as were approved by the Local Ethics Committee for Animal Experimentation in Poznań (Protocol No. 80/2013). The studies were performed on syngenic, healthy, adult, male Wistar rats with an average body weight of 250 ± 30 g. The animals were maintained on a 12-hour light-dark cycle in a humidity- (50 ± 5%) and temperature- (21 ± 1°C) controlled room, with a ventilation rate of 12 air changes per hour, and had free access to water and food (standard laboratory diet, Labofeed B, Feed Factory “Morawski”, Żurawia, Poland). All experiments were carried out at the same time in the morning. After two weeks of acclimatization and observation, the animals were divided into two experimental groups of 40 animals each: one group of rats with normoglycemia receiving 1.0 mL of 0.1 mol/L fresh cold citrate buffer (pH 4.5), intraperitoneally 72 h before sacrifice and the group of rats with hyperglycemia induced by an intraperitoneal injection of STZ (Sigma-Aldrich, Inc., St. Louis, MO, USA), 65 mg/kg body weight (21), into 12 h-fasted rats, 72 h before sacrifice. STZ was dissolved in 1.0 mL of 0.1 mol/L citrate buffer (pH 4.5). Hyperglycemia was verified by measuring fasting plasma glucose using Liquick Cor-GLUCOSE kit (Cormay, S.A., Lublin, Poland), and diagnosed by glucose concentration >15 mmol/L. Plasma insulin concentration was measured by radioimmunoassay, using Insulin RIA kit (Linco Research, Inc., St. Charles, MO, USA). Each group was divided into five subgroups. Control subgroup (Control, n = 8) received 1.0 mL of 0.15 mol/L NaCl, intraperitoneally, 2 hours and 1 hour before sacrifice. Captopril subgroup (CAP, n = 8) received 1.0 mL of 0.15 mol/L NaCl, intraperitoneally, 2 hours, and captopril (Sigma-Aldrich, Inc., St. Louis, MO, USA), 3 mg/kg body weight (22), intraperitoneally, 1 hour before sacrifice. Captopril plus antagonists subgroup (CAP+ANT, n = 8) received HOE-140 (D-Arg-[Hyp^3^,Thi^5^,Dtic^7^-Oic^8^]-bradykinin), a B2Rs antagonist (Sigma-Aldrich, Inc., St. Louis, MO, USA), 300 μg/kg body weight (23), subcutaneously, and desArg^9^[Leu^8^]-bradykinin, a B1Rs antagonist (Sigma-Aldrich, Inc.), 300 μg/kg body weight (24), subcutaneously, 2 hours, and captopril, intraperitoneally, 3 mg/kg body weight 1 hour before sacrifice. Enalapril subgroup (ENP, n = 8) received 1.0 mL of 0.15 mol/L NaCl, intraperitoneally, 2 hours, and enalapril (Sigma-Aldrich, Inc.), 1 mg/kg body weight (25), intraperitoneally, 1 hour before sacrifice. Enalapril plus antagonists subgroup (ENP+ANT, n = 8) received HOE-140 and desArg^9^[Leu^8^]-bradykinin 2 hours, and enalapril 1 hour before sacrifice. Drug doses and the administration route were the same as respectively in the CAP+ANT and ENP subgroup. Captopril, enalapril, and B1 and B2 receptor antagonists were dissolved in 0.15 mol/L NaCl. Rats were anesthetized with a single intraperitoneal injection of ketamine hydrochloride (Bioketan, Vetoquinol Biowet, Sp. z o.o., Gorzów Wlkp., Poland), 100 mg/kg body weight and xylazine hydrochloride (Xylapan, Vetoquinol Biowet, Sp. z o.o., Gorzów Wlkp., Poland), 10 mg/kg body weight. After sampling the rats were killed by exsanguination (Figure 1).

### Tissue sampling and analyses

Blood samples were taken from the anaesthetized rats by the puncture of the right ventricle, collected in heparinized tubes, and prepared as described by the kits’ manufacturers and using Frew procedure (26).

The concentration of plasma malondialdehyde was determined using assay kit BIOXYTECH^®^ MDA-586 (OXIS International, Inc., Beverly Hills, CA, USA). The method is based on the reaction of N-methyl-2-phenylindole (NMPI) with MDA at 45°C in the presence of HCl. One molecule of MDA reacts with two molecules of NMPI to create a stable chromophore form with a maximum absorbance at 586 nm. The absorbance was measured spectrophotometrically (Beckman Coulter, Inc., Brea, CA, USA) at 586 nm. MDA concentration was expressed as µmol/L.

The concentration of plasma hydrogen peroxide (H_2_O_2_) was determined spectrophotometrically using the Frew procedure (21). The reagent solution (100 mL) contained 0.234 g of phenol, 0.1 g of 4-aminoantipyrine, 1.0 mL of 0.1 mol/L phosphate buffer (pH 6.9), about 2 × 10^−8^ horseradish peroxidase (HRP), and about 2.5 µmol/L H_2_O_2_. H_2_O_2_ coupled with 4-aminoantipyrine, and phenol, in the presence of peroxidase, to yield a chromogen with maximum absorbance at 505 nm. H_2_O_2_ concentration was expressed as mmol H_2_O_2_/mL.

Antioxidative enzymes activities were measured in the erythrocyte fractions. SOD activity was determined using assay kit BIOXYTECH^®^ SOD-525 (OXIS International, Inc.). The method is based on the SOD-catalyzed increase of autooxidation of 5,6,6a, 11b-tetrahydro-3,9,10-trihydroxybenzo[c]-fluorene to a chromophore form with maximum absorbance at 525 nm. The absorbance changes were determined spectrophotometrically at 525 nm at 10-second intervals for one minute. SOD activity was expressed as U/mg Hb.

CAT activity was determined using assay kit BIOXYTECH^®^ Catalase-520 (OXIS International, Inc.). The method is a two-step procedure based on the assumption that the rate of dismutation of H_2_O_2_ to water, and molecular oxygen is proportional to the concentration of CAT. In the first step the sample containing CAT was subjected to one-minute incubation in a known concentration of H_2_O_2_. After incubation the reaction was quenched with sodium azide. In the second step the amount of H_2_O_2_ remaining in the sample was determined by the coupling reaction of H_2_O_2_ with 4-aminophenazone (4-aminoantipyrene, AAP), and 3,5-dichloro-2-hydroksybenzenesulfonic acid (DHBS) in the presence of HRP, acting as a catalyst. The absorbance of the resulting colored product, quinoneimine, was measured spectrphotometrically at 520 nm. CAT activity was expressed as U/mg Hb.

GPx activity was measured using assay kit RANSEL RS 505 (Randox, Crumlin, UK). In this method GPx catalyzes the oxidation of glutathione by cumene hydroperoxide. In the presence of glutathione reductase and NADPH the oxidized glutathione is converted to the reduced form with a concominant oxidation of NADPH to NADP^+^. The production of NADP^+^ was measured spectrophotometrically at 340 nm. GPx activity was expressed as U/g Hb.

### Statistical analysis

Data are expressed as a mean ± standard deviation (SD) obtained from n = 8 rats. The normality of the data distribution was tested using the Shapiro-Wilk test (STATISTICA 12.5., StatSoft Polska, Kraków, Poland, *P* > 0.050 as normal). Statistical significance was determined by using the one-way ANOVA followed by the Tukey post-hoc analysis (STATISTICA 12.5.) or the Welch ANOVA and the Games-Howell post-hoc procedure for multiple comparisons (PQStat 1.6.2., PQStat Software, Poznań, Poland). Differences were considered to be statistically significant at *P* < 0.050.

## Results

**Figure 2:**
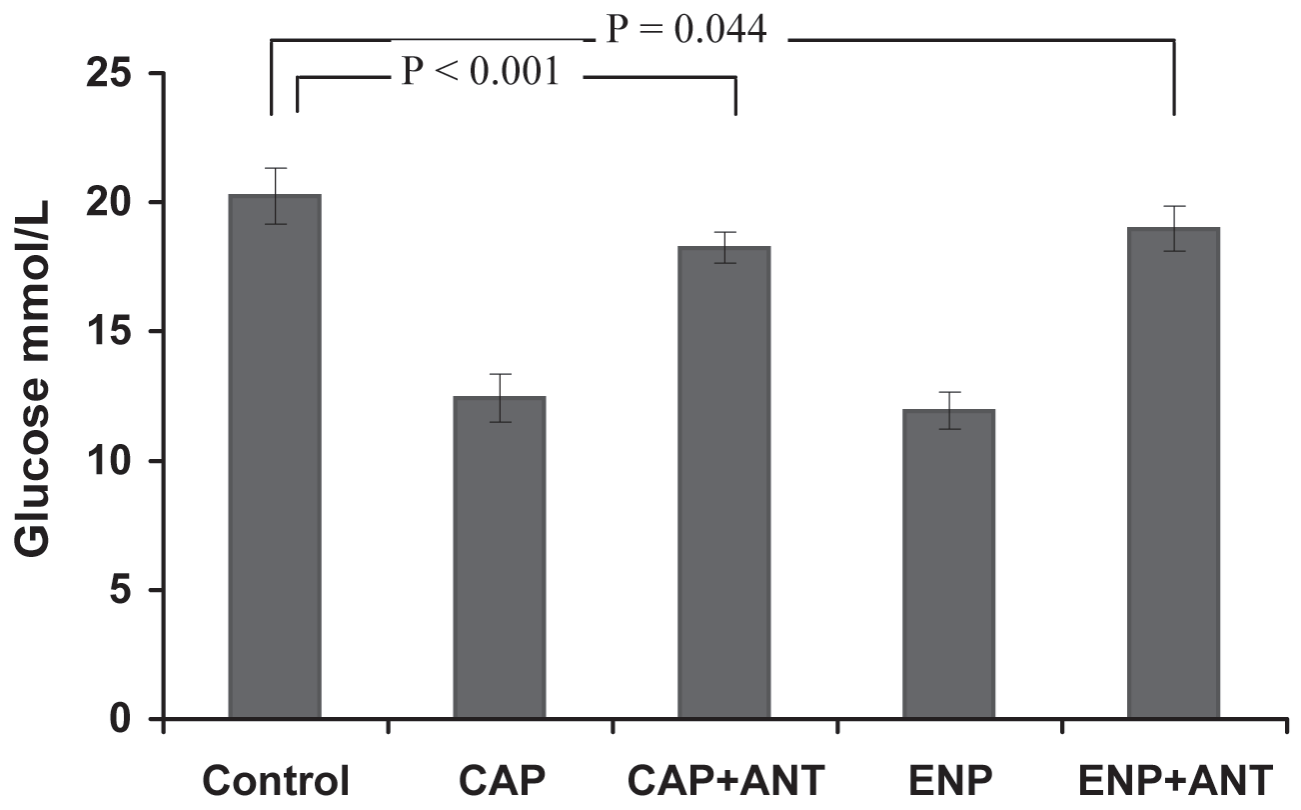
Glucose concentration (mmol/L) in plasma of hyperglycemic rats after captopril (CAP), enalapril (ENP), and bradykinin receptor antagonists (ANT) administration. Mean ± SD, n = 8 per group.

**Figure 3:**
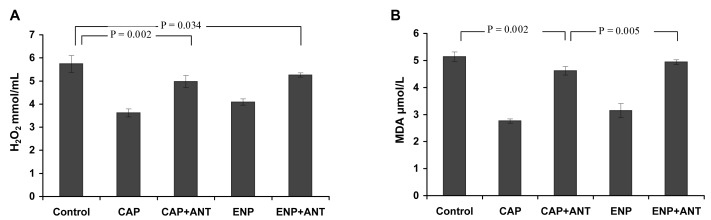
Oxidative stress markers concentration in plasma of hyperglycemic rats after captopril (CAP), enalapril (ENP), and bradykinin receptor antagonists (ANT) administration. (**A)**: hydrogen peroxide (H_2_O_2_, mmol/mL); (**B)**: malondialdehyde (MDA, µmol/L). Mean ± SD, n = 8 per group.

**Figure 4:**
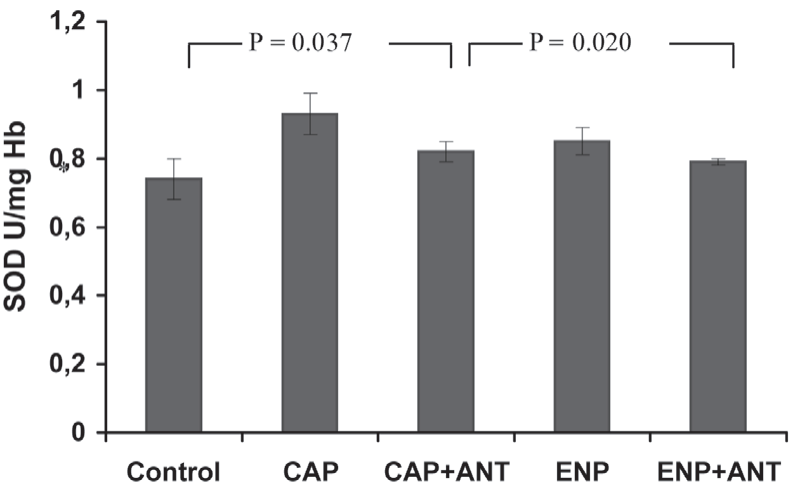
Superoxide dismutase activity (SOD, U/mg Hb) in erythrocytes of hyperglycemic rats after captopril (CAP), enalapril (ENP), and bradykinin receptor antagonists (ANT) administration. Mean ± SD, n = 8 per group.

STZ administration increased plasma glucose (*P* < 0.001) and decreased plasma insulin (*P* < 0.001) in comparison to the normoglycemic group (Table 1). Captopril and enalapril significantly decreased glucose concentration in hyperglycemic rats (*P* < 0.001) and significantly increased insulin concentration (*P* = 0.002) in comparison to the control hyperglycemic subgroup (Table 1, Figure 2). Captopril and enalapril also significantly decreased plasma glucose (*P* < 0.001 and *P* = 0.044, respectively) in hyperglycemic rats pre-treated with HOE-140 and desArg^9^[Leu^8^]-bradykinin in comparison to the control hyperglycemic subgroup (Table 1, Figure 2). H_2_O_2_ and MDA concentration in plasma of hyperglycemic rats was significantly increased (*P* < 0.001) in comparison to the normoglycemic group (Table 2). Captopril and enalapril in hyperglycemic rats significantly decreased H_2_O_2_ and MDA concentration (*P* < 0.001) in comparison to the control hyperglycemic subgroup (Table 2, Figure 3 A, B). In the presence of HOE-140 and desArg^9^[Leu^8^]-bradykinin, both captopril and enalapril significantly decreased H_2_O_2_ concentration in rats with hyperglycemia in comparison to the control hyperglycemic subgroup (*P* = 0.002 and *P* = 0.034, respectively), but only captopril significantly reduced MDA concentration in rats treated with bradykinin receptor antagonists (*P* < 0.001) (Table 2, Figure 3 A, B). The activity of SOD, CAT, and GPx in erythrocytes of hyperglycemic rats was significantly decreased *(P <* 0.001) in comparison to the normoglycemic group (Table 3). In hyperglycemic rats treated with captopril and enalapril, SOD (*P* = 0.006), CAT (only captopril, *P* < 0.001), and GPx (*P* < 0.001) were significantly increased in comparison to the control hyperglycemic subgroup (Table 3). Captopril in hyperglycemic rats receiving B1 and B2 receptor antagonists, desArg^9^[Leu^8^]-bradykinin, and HOE-140, significantly increased SOD (*P* = 0.037) in comparison to the control hyperglycemic subgroup (Table 3, Figure 4).

**Table 1:** Glucose (mmol/L) and insulin (mU/L) concentration in plasma of normoglycemic and hyperglycemic rats after captopril (CAP), enalapril (ENP), and bradykinin receptor antagonists (ANT) administration*†‡

	Control	CAP	CAP+ANT	ENP	ENP+ANT
**Glucose (mmol/L)**					
Normoglycemia	5.93 ± 0.22	5.81 ± 0.31	6.00 ± 0.23	5.84 ± 0.41	5.92 ± 0.34
*P^†^*	0.234	0.137	0.737	0.125	0.094
Hyperglycemia	20.24 ± 1.08	12.42 ± 0.93^§^	18.24 ± 0.61^§║^	11.93 ± 0.71^§^	18.98 ± 0.87^§¶^
*P^†^*	0.098	0.164	0.760	0.298	0.863
**Insulin (mU/L)**					
Normoglycemia	46.94 ± 1.24	46.63 ± 2.01	45.93 ± 1.69	46.49 ± 1.81	46.83 ± 1.48
*P^†^*	0.209	0.479	0.211	0.244	0.283
Hyperglycemia	2.53 ± 0.58	3.82 ± 0.23^§^	2.64 ± 0.31^║^	3.71 ± 0.21^§^	2.64 ± 0.21^¶^
*P^†^*	0.423	0.320	0.461	0.375	0.172

*Data are expressed as a mean ± standard deviation, n = 8 animals per group.

†Shapiro-Wilk test *P* values.

‡All results were statistically significant in comparison to the normoglycemic group (*P* < 0.001).

§*P* < 0.001 (ENP+ANT, *P* = 0.044) vs hyperglycemic control.

║*P* < 0.001 vs CAP.

¶*P* < 0.001 vs ENP.

**Table 2:** Hydrogen peroxide (H_2_O_2_, mmol/mL) and malondialdehyde (MDA, µmol/L) concentration in plasma of normoglycemic and hyperglycemic rats after captopril (CAP), enalapril (ENP), and bradykinin receptor antagonists (ANT) administration*†‡

	Control	CAP	CAP+ANT	ENP	ENP+ANT
**H_2_O_2_ (mmol/mL)**					
Normoglycemia	2.10 ± 0.18	1.69 ± 0.09	1.92 ± 0.10	1.81 ± 0.29	1.90 ± 0.46
*P^†^*	0.629	0.155	0.408	0.293	0.407
Hyperglycemia	5.74 ± 0.36	3.62 ± 0.17^§^	4.97 ± 0.26^§║^	4.09 ± 0.14^§║^	5.26 ± 0.09^§¶^
*P ^†^*	0.251	0.150	0.121	0.195	0.176
**MDA (µmol/L)**					
Normoglycemia	3.77 ± 0.31	1.84 ± 0.21^§^	3.62 ± 0.51^║^	3.63 ± 0.37^║^	4.04 ± 0.14
*P^†^*	0.176	0.159	0.221	0.097	0.095
Hyperglycemia	5.14 ± 0.17	2.76 ± 0.08^§^	4.62 ± 0.16^§║^	3.14 ± 0.26^§║^	4.94 ± 0.09^¶**^
*P^†^*	0.708	0.401	0.113	0.366	0.349

*Data are expressed as a mean ± standard deviation (SD), n = 8 animals per group.

†Shapiro-Wilk test *P* value.

‡All results were statistically significant in comparison to the normoglycemic group (*P* < 0.001).

§*P* = 0.002 (ENP+ANT, *P* = 0.034) vs respectively normoglycemic or hyperglycemic control.

║*P* < 0.001 vs CAP.

¶*P* < 0.001 vs ENP.

**P = 0.005 vs CAP+ANT.

**Table 3:** Superoxide dismutase (SOD, U/mg Hb), catalase (CAT, U/mg Hb), and glutathione peroxidase (GPx, U/g Hb) activity in erythrocytes of normoglycemic and hyperglycemic rats after captopril (CAP), enalapril (ENP), and bradykinin receptor antagonists (ANT) administration*†‡

	Control	CAP	CAP+ANT	ENP	ENP+ANT
**SOD (U/mg Hb)**					
Normoglycemia	1.36 ± 0.07	1.34 ± 0.08	1.38 ± 0.08	1.36 ± 0.05	1.38 ± 0.07
*P^†^*	0.100	0.397	0.203	0.153	0.109
Hyperglycemia	0.74 ± 0.06	0.93 ± 0.06^§^	0.82 ± 0.03^§║^	0.85 ± 0.04^§║^	0.79 ± 0.01^¶**^
*P^†^*	0.085	0.096	0.234	0.358	0.353
**CAT (U/mg Hb)**					
Normoglycemia	108.62 ± 4.42	106.14 ± 2.47	107.59 ± 5.17	107.11 ± 4.58	108.61 ± 3.40
*P^†^*	0.139	0.530	0.198	0.079	0.107
Hyperglycemia	90.82 ± 5.80	101.73 ± 4.70^§^	95.11 ± 4.80	97.36 ± 6.09	92.14 ± 3.10
*P^†^*	0.203	0.075	0.324	0.069	0.342
**GPx (U/g Hb)**					
Normoglycemia	240.12 ± 19.18	244.49 ± 20.51	232.18 ± 19.67	242.11 ± 18.53	236.18 ± 18.41
*P^†^*	0.103	0.143	0.146	0.311	0.677
Hyperglycemia	162.11 ± 11.78	214.33 ± 15.13^§^	176.55 ± 13.21^║^	200.37 ± 10.36^§^	163.92 ± 10.02^¶^
*P^†^*	0.082	0.088	0.287	0.114	0.274

*Data are expressed as a mean ± standard deviation (SD), n = 8 animals per group.

† Shapiro-Wilk test *P* value.

‡All results were statistically significant in comparison to the normoglycemic group (*P* < 0.001; CAT/CAP, *P* = 0.034).

§*P* < 0.001 (SOD/CAP+ANT, *P* = 0.037; SOD/ENP, *P* = 0.006) vs hyperglycemic control.

║*P* < 0.001 (SOD/ENP, *P* = 0.037) vs CAP.

¶*P* = 0.006 vs ENP.

**P = 0.020 vs CAP+ANT.

## Discussion

We observed that treatment with captopril or enalapril together with bradykinin receptor antagonists decreased the concentration of oxidative stress markers (H_2_O_2_ and MDA) and increased antioxidative activity in hyperglycemic rats. This may suggest that antioxidative properties of ACEIs under hyperglycemic conditions may be independent of bradykinin activity. Presumably, this effect is associated not only with the regulation of RAS, but with an influence independent of it. The main mechanism by which ACEIs prevent the development of oxidative stress in hyperglycemic conditions is associated with the activation of bradykinin-nitric oxide-prostacyclin system. ACE inhibition increases the production of bradykinin, which stimulates release of vasodilating substances, such as nitric oxide (NO) and prostacyclin (27). An improvement of blood flow increases oxygen supply, oxygen consumption, insulin secretion, insulin, and glucose delivery to the insulin-sensitive tissues, and facilitates insulin signaling at the cellular level. This, in turn, reduces serum glucose level and ROS production (28,29).

Several studies have demonstrated that the beneficial effects of ACEIs in diabetic patients are attenuated by B2Rs antagonists, suggesting that the protective effects of ACEIs are mediated by bradykinin and B2Rs signaling (19,20,30,31). On the other hand, activation of kinin B1Rs enhances O2^●−^ production, while prolonged blockade or even lack of B1Rs reduces oxidative stress, metabolic complications, and cardiovascular abnormalities in hyperglycemic animals (14,32). This suggests that both types of kinin receptors may be involved in the regulation of redox state in experimental DM. Recent studies indicate that ACEIs directly, as allosteric agonists, or indirectly, via inactivation of ACE, affect B1R and B2R signaling and regulate oxidant/antioxidant status (15,33). Our results showed that in the presence of B1R and B2R antagonists, ACEIs still decrease oxidative stress markers in the hyperglycemic rats. Taken together, the reduction of the antioxidant ACEIs activity caused by B2Rs antagonists may be due to the limited action of bradykinin, however, it cannot be ruled out that ACEIs directly enhance antioxidative activity and that both mechanisms (bradykinin-mediated and bradykinin-independent) play a role. Tasdemir et al (34) reported reduced necrosis/total heart zone ratio in rats with myocardial infraction after captopril administration, and a non-significant decrease of this ratio in captopril+B2R antagonist group. They concluded that non-significant decrease in the protective effects of captopril by B2R blocker indicates the existence of the routes independent of bradykinin action. This route may be possibly associated with the activation of non-classic RAS axis and regulation of the prooxidative enzymes activity. It is known that RAS consists of two separate but reciprocally controlled axes: the classic ACE/ANG-II/AT1 axis, responsible for the vasoconstricting/proliferative effects, and ACE2/ANG- (1-7)/Mas axis, which counter-regulates ACE activity by reducing ANG-II bioavailability and simultaneously increases the formation of a vasoprotective/antiproliferative peptide, ANG- (1-7). ACE2 is an endogenous regulator of RAS, which maintains the balance between these two axes (35). ACE2 could also be involved in the regulation of oxidative stress, since ACE2 knockout mice demonstrate elevated concentration of oxidative stress markers (36,37). ACEIs inhibit ACE activity but do not attenuate or even increase ACE2 activity (38,39), which, in turn, increases ANG- (1-7) concentration (40-44). Existing reports indicate a protective effect of ANG- (1-7) in the suppression of oxidative stress regulated by NADPH oxidase, which is considered to be a major source of ROS in the diabetic environment (45,46). Moreover, ACEIs are also remarkably effective in the recoupling of eNOS. It should be noted that the uncoupled form of this enzyme is an important source of superoxide anions under pathological conditions (47-51).

It is well known that excessive glucose can contribute to the development of oxidative stress (2-4). ACEIs improve insulin sensitivity and glucose metabolism, and reduce plasma glucose both under experimental conditions and in patients with DM (28). Probably this effect is mostly dependent on bradykinin, since it can be considerably blocked by bradykinin receptor antagonists (52). Our findings provide yet another possible mechanism. As we observed, both captopril and enalapril can reduce glucose concentration in rats treated with B1Rs and B2Rs antagonists, which indicates bradykinin-independent pathway. As we mentioned before, ACEIs increase ANG- (1-7) concentration (40,43). ANG- (1-7) stimulates adiponectin release, thereby improving insulin sensitivity and glucose utilization in the peripheral tissues (42,53). Moreover, ANG- (1-7) is able to modulate insulin signaling pathway through the activation of IRS-1, JAK2, and Akt kinase in the main target tissues for insulin (54,55). Also, ACEIs can probably regulate glucose concentration by affecting the production and the activity of glucose transporters (42,56,57). To summarize, ACEIs, by intensifying glucose uptake and reducing plasma glucose, may play a protective, antioxidative role in hyperglycemic subjects.

We also noted that captopril, in contrast to enalapril, reduced MDA concentration in the presence of B1Rs and B2Rs antagonists, which may indicate its stronger antioxidant properties. Captopril is well known to have a free radical scavenging property, as well as reducing potency in various pathological conditions associated with oxidative stress. The beneficial antioxidant properties of captopril could probably be related to the presence of the unblocked sulphydryl group in the drug molecule (58). Sulfhydryl group as a metal chelator, radical quencher, and component of the thiol/disulfide redox buffer has been recognized as a potential free radical scavenging factor (59). Interestingly, in the organism only a part of captopril binds with ACE, whereas the rest exists in an adsorbed form or as disulfides formed by the reaction with endogenous sulphydryl-containing compounds, such as glutathione or plasma proteins. Disulfides are considered as a drug depot, thus captopril may function as a recyclable antioxidant (60). In the interpretation of our results, a few limitations should be considered. First, STZ-induced experimental DM is an insulin-dependent diabetes (type 1), therefore it may not be an appropriate model to evaluate the role of ACEIs and B1Rs and B2Rs antagonists in diabetes type 2. Second, we limited our study to acute hyperglycemia and a single dose of ACEIs. It should be noted, however, that acute ACE inhibition reduces the ANG-II concentration, but prolonged ACEIs treatment leads to ACE up-regulation and increases the ANG-II/ANG-I ratio (61). ANG-II is a potentially prooxidative agent (62), and may change the oxidant/antioxidant status, thus, chronic ACEIs therapy may have a different effect on oxidative stress than short-term use of these drugs.

In conclusion, ACEIs are able to reduce oxidative stress intensity in hyperglycemic conditions. Bradykinin appears to be the main factor mediating antioxidative properties of ACEIs, although our results suggest the existence of additional effect of ACEIs in hyperglycemic conditions, which is not related to the bradykinin mediation and the structure of the drug molecule. Further studies are necessary to investigate the detailed mechanisms of ACEIs action.

## References

[1] Monnier L, Mas E, Ginet C, Michel F, Villon L, Cristol JP (2006). Activation of oxidative stress by acute glucose fluctuations compared with sustained chronic hyperglycemia in patients with type 2 diabetes.. JAMA.

[2] de M Bandeira S, da Fonseca LJ, da S Guedes G, Rabelo LA, Goulart MO, Vasconcelos SM (2013). Oxidative stress as an underlying contributor in the development of chronic complications in diabetes mellitus.. Int J Mol Sci.

[3] Evans JL, Goldfine ID, Maddux BA, Grodsky GM (2002). Oxidative stress and stress-activated signaling pathways: a unifying hypothesis of type 2 diabetes.. Endocr Rev.

[4] Wohaieb SA, Godin DV (1987). Alterations in free radical tissue-defense mechanisms in streptozocin-induced diabetes in rat. Effects of insulin treatment.. Diabetes.

[5] McGuire DK, Winterfield JR, Rytlewski JA, Ferrannini E (2008). Blocking the renin-angiotensin-aldosterone system to prevent diabetes mellitus.. Diab Vasc Dis Res.

[6] Andraws R, Brown DL (2007). Effect of inhibition of the renin-angiotensin system on development of type 2 diabetes mellitus (meta-analysis of randomized trials).. Am J Cardiol.

[7] Funakawa S, Okahara T, Imanishi M, Komoro T, Yamamoto K, Tochio Y (1983). Renin-angiotensin system and prostacyclin biosynthesis in streptozotocin diabetic rats.. Eur J Pharmacol.

[8] Lieberman J, Sastre A (1980). Serum angiotensin-converting enzyme: Elevation in diabetes mellitus.. Ann Intern Med.

[9] Abuissa H, Jones PG, Marso SP, O’Keefe JJH (2005). Angiotensin-converting enzyme inhibitors or angiotensin receptor blockers for prevention of type 2 diabetes: a meta-analysis of randomized clinical trials.. J Am Coll Cardiol.

[10] Münzel T, Keaney JF (2001). Are ACE inhibitors a “magic bullet” against oxidative stress?. Circulation.

[11] Hornig B, Kohler C, Drexler H (1997). Role of bradykinin in mediating vascular effects of angiotensin-converting enzyme inhibitors in humans.. Circulation.

[12] Couture R, Girolami JP (2004). Putative roles of kinin receptors in the therapeutic effects of angiotensin 1-converting enzyme inhibitors in diabetes mellitus.. Eur J Pharmacol.

[13] Ni A, Chao L, Chao J (1998). Transcription factor nuclear factor kappaB regulates the inducible expression of the human B1 receptor gene in inflammation.. J Biol Chem.

[14] Dias JP, Talbot S, Sénécal J, Carayon P, Couture R (2010). Kinin B1 receptor enhances the oxidative stress in a rat model of insulin resistance: outcome in hypertension, allodynia and metabolic complications.. PLoS ONE.

[15] Erdös EG, Tan F, Skidgel RA (2010). Angiotensin I-converting enzyme inhibitors are allosteric enhancers of kinin B1 and B2 receptor function.. Hypertension.

[16] Chopra M, Beswick H, Clapperton M, Dargie HJ, Smith WE, McMurray J (1992). Atioxidant effects of angiotensin-converting enzyme (ACE) inhibitors: free radical and oxidant scavenging are sulfhydryl dependent, but lipid peroxidation is inhibited by both sulfhydryl- and nonsulfhydryl-containing ACE inhibitors.. J Cardiovasc Pharmacol.

[17] Evangelista S, Manzini S (2005). Antioxidant and cardioprotective properties of the sulphydryl angiotensin-converting enzyme inhibitor zofenopril.. J Int Med Res.

[18] Chin BS, Langford NJ, Nuttall SL, Gibbs CR, Blann AD, Lip GY (2003). Anti-oxidative properties of beta-blockers and angiotensin-converting enzyme inhibitors in congestive heart failure.. Eur J Heart Fail.

[19] Manolis AJ, Marketou ME, Gavras I, Gavras H (2010). Cardioprotective properties of bradykinin: role of the B(2) receptor.. Hypertens Res.

[20] Oeseburg H, Iusuf D, van der Harst P, van Gilst WH, Henning RH, Roks AJ (2009). Bradykinin protects against oxidative stress-induced endothelial cell senescence.. Hypertension.

[21] Bell RH, Hye RJ (1983). Animal models of diabetes mellitus: physiology and pathology.. J Surg Res.

[22] Kopecky RT, Thomas FD, McAfee JG (1987). Furosemide augments the effects of captopril on nuclear studies in renovascular stenosis.. Hypertension.

[23] Madeddu P, Anania V, Parpaglia PP, Demontis MP, Varoni MV, Pisanu G (1992). Effects of Hoe 140, a bradykinin B2-receptor antagonist, on renal function in conscious normotensive rats.. Br J Pharmacol.

[24] Zuccollo A, Navarro M, Catanzaro O (1996). Effects of B1 and B2 kinin receptor antagonists in diabetic mice.. Can J Physiol Pharmacol.

[25] D'Souza RS, Bhounsule SA, Dhume VG (1990). Comparison of the effects of captopril and enalapril on oxyphenbutazone and ethanol-induced gastric lesions in rats.. Indian J Physiol Pharmacol.

[26] Frew JE, Jones P, Scholes G (1983). Spectrophotometric determination of hydrogen peroxide and organic hydropheroxides at low concentrations in aqueous solution.. Anal Chim Acta.

[27] Vanhoutte PM, Boulanger CM, Illiano SC, Nagao T, Vidal M, Mombouli JV (1993). Endothelium-dependent effects of converting-enzyme inhibitors.. J Cardiovasc Pharmacol.

[28] Scheen AJ (2004). Prevention of type 2 diabetes mellitus through inhibition of the renin-angiotensin system.. Drugs.

[29] Eshaq RS, Wright WS, Harris NR (2014). Oxygen delivery, consumption, and conversion to reactive oxygen species in experimental models of diabetic retinopathy.. Redox Biol..

[30] Buléon M, Allard J, Jaafar A, Praddaude F, Dickson Z, Ranera MT (2008). *Pharmacological* blockade of B2-kinin receptor reduces renal protective effect of angiotensin-converting enzyme inhibition in db/db mice model.. Am J Physiol Renal Physiol.

[31] Kakoki M, Kizer CM, Yi X, Takahashi N, Kim HS, Bagnell CR (2006). Senescence-associated phenotypes in Akita diabetic mice are enhanced by absence of bradykinin B2 receptors.. J Clin Invest.

[32] Westermann D, Walther T, Savvatis K, Escher F, Sobirey M, Riad A (2009). Gene deletion of the kinin receptor B1 attenuates cardiac inflammation and fibrosis during the development of experimental diabetic cardiomyopathy.. Diabetes.

[33] Mastan Rao Y, Aparna Lakshmi I, Saroja M (2011). Effect of ACE inhibitors on antioxidant status in streptozotocin induced diabetic rats.. Asian J Pharm Clin Res..

[34] Tasdemir S, Parlakpinar H, Acet A (2013). Role of bradykinin in the cardioprotective effects of captopril and angiotensin II receptor blockers (AT1, AT2) on myocardial ischemia-reperfusion injury in rats.. Health Med.

[35] Guang C, Phillips RD, Jiang B, Milani F (2012). Three key proteases–angiotensin-I-converting enzyme (ACE), ACE2 and renin–within and beyond the renin-angiotensin system.. Arch Cardiovasc Dis..

[36] Wysocki J, Ortiz-Melo DI, Mattocks NK, Xu K, Prescott J, Evora K (2014). ACE2 deficiency increases NADPH-mediated oxidative stress in the kidney.. Physiol Rep..

[37] Song B, Jin H, Yu X, Zhang Z, Yu H, Ye J (2013). Angiotensin-converting enzyme 2 attenuates oxidative stress and VSMC proliferation via the JAK2/STAT3/SOCS3 and profilin-1/MAPK signaling pathways.. Regul Pept.

[38] Tipnis SR, Hooper NM, Hyde R, Karran E, Christie G, Turner AJ (2000). A human homolog of angiotensin-converting enzyme: cloning and functional expression as a captopril-insensitive carboxypeptidase.. J Biol Chem.

[39] Malfitano C, De Angelis K, Fernandes T, Wichi RB, Rosa K, Pazzine M (2012). Low-dose enalapril reduces angiotensin II and attenuates diabetic-induced cardiac and autonomic dysfunctions.. J Cardiovasc Pharmacol.

[40] Lavrentyev EN, Malik KU (2009). High glucose-induced Nox1-derived superoxides downregulate PKC-*β*II, which subsequently decreases ACE2 expression and ANG(1-7) formation in rat VSMCs.. Am J Physiol Heart Circ Physiol.

[41] Lavrentyev EN, Malik KU (2008). High glucose (HG)-induced angiotensin-converting enzyme 2 (ACE2) and angiotensin (1–7) [Ang (1–7)] decrease is prevented by captopril, losartan and insulin in rat vascular smooth muscle cells (VSMCs).. FASEB J.

[42] Frantz ED, Crespo-Mascarenhas C, Barreto-Vianna AR, Aguila MB, Mandarim-de-Lacerda CA (2013). Renin-angiotensin system blockers protect pancreatic islets against diet-induced obesity and insulin resistance in mice.. PLoS ONE.

[43] Santos RA, Ferreira AJ, Simőes E, Silva AC (2008). Recent advances in the angiotensin-converting enzyme 2-angiotensin(1-7)-Mas axis.. Exp Physiol.

[44] Echeverría-Rodríguez O, Del Valle-Mondragón L, Hong E (2014). Angiotensin 1-7 improves insulin sensitivity by increasing skeletal muscle glucose uptake in vivo.. Peptides.

[45] Liu C, Lv XH, Li HX, Cao X, Zhang F, Wang L (2012). Angiotensin-(1-7) suppresses oxidative stress and improves glucose uptake via Mas receptor in adipocytes.. Acta Diabetol.

[46] Zhang K, Meng X, Li D, Yang J, Kong J, Hao P (2015). Angiotensin(1-7) attenuates the progression of streptozotocin-induced diabetic renal injury better than angiotensin receptor blockade.. Kidney Int.

[47] Förstermann U, Münzel T (2006). Endothelial nitric oxide synthase in vascular disease. From marvel to menace.. Circulation.

[48] Oak JH, Cai H (2007). Attenuation of angiotensin II signaling recouples eNOS and inhibits nonendothelial NOX activity in diabetic mice.. Diabetes.

[49] Tabbi-Anneni I, Buchanan J, Cooksey RC, Abel ED (2008). Captopril normalizes insulin signaling and insulin-regulated substrate metabolism in obese (*ob/ob*) mouse hearts.. Endocrinology.

[50] Yamazaki T, Tanimoto M, Gohda T, Ohara I, Hagiwara S, Murakoshi M (2009). Combination effects of enalapril and losartan on lipid peroxidation in the kidneys of KK-Ay/Ta mice.. Nephron Exp Nephrol.

[51] de Cavanagh EM, Inserra F, Ferder L, Fraga CG (2000). Enalapril and captopril enhance glutathione-dependent antioxidant defenses in mouse tissues.. Am J Physiol Regul Integr Comp Physiol.

[52] Kudoh A, Matsuki A (2000). Effects of angiotensin-converting enzyme inhibitors on glucose uptake.. Hypertension.

[53] Santos SH, Braga JF, Mario EG, Pôrto LC, Rodrigues-Machado Mda G, Murari A (2010). Improved lipid and glucose metabolism in transgenic rats with increased circulating angiotensin-(1-7).. Arterioscler Thromb Vasc Biol.

[54] Prasannarong M, Santos FR, Henriksen EJ (2012). ANG-(1-7) reduces ANG II-induced insulin resistance by enhancing Akt phosphorylation via a Mas receptor-dependent mechanism in rat skeletal muscle.. Biochem Biophys Res Commun.

[55] Giani JF, Gironacci MM, Muńoz MC, Peńa C, Turyn D, Dominici FP (2007). Angiotensin-(1–7) stimulates the phosphorylation of JAK2, IRS-1 and Akt in rat heart in vivo: role of the AT1 and Mas receptors.. Am J Physiol Heart Circ Physiol.

[56] Katayama S, Inaba M, Maruno Y, Morita T, Awata T, Oka Y (1997). Glucose intolerance in spontaneously hypertensive and Wistar-Kyoto rats: enhanced gene expression and synthesis of skeletal muscle glucose transporter 4.. Hypertens Res.

[57] Wakisaka M, Yoshinari M, Nakamura S, Asano T, Sonoki K, Shi AH (1999). Suppression of sodium-dependent glucose uptake by captopril improves high glucose-induced morphological and functional changes of cultured bovine retinal pericytes.. Microvasc Res.

[58] de Cavanagh EM, Insera F, Ferder L, Romano L, Ercole L, Fraga CG (1995). Superoxide dismutase and glutathione peroxidase activities are increased by enalapril and captopril in mouse liver.. FEBS Lett.

[59] Petrov L, Atanassova M, Alexandrova A (2012). Comparative study of the antioxidant activity of some thiol-containing substances.. Cent Eur J Med..

[60] Chopra M, Scott N, McMurray J, McLay J, Bridges A, Smith WE (1989). Captopril: a free radical scavenger.. Br J Clin Pharmacol.

[61] Danser AHJ. Renin-angiotensin system – plasma versus tissues. In: Unger T, Schӧlkens BA, editors. Angiotensin Vol.1. Berlin Heidelberg: Springer-Verlag; 2004. p.129-148.

[62] Singh VP, Le B, Khode R, Baker KM, Kumar R (2008). Intracellular angiotensin II production in diabetic rats is correlated with cardiomiocyte apoptosis, oxidative stress, and cardiac fibrosis. Diabetes.

